# (2*Z*)-2-Fluoro-*N*-{4-[5-(4-fluoro­phen­yl)-2-methyl­sulfanyl-1*H*-imidazol-4-yl]-2-pyrid­yl}-3-phenyl­acrylamide

**DOI:** 10.1107/S1600536809051010

**Published:** 2009-11-28

**Authors:** Roland Selig, Dieter Schollmeyer, Thomas Stegmiller, Wolfgang Albrecht, Stefan Laufer

**Affiliations:** a Eberhard-Karls-University Tübingen, Auf der Morgenstelle 8, 72076 Tübingen, Germany; b UniversitätMainz, Institut für Organische Chemie, Duesbergweg 10-14, 55099 Mainz, Germany; c c-a-i-r biosciences GmbH, Paul-Ehrlich-Str. 15, 72076 Tübingen, Germany

## Abstract

The asymmetric unit of the title compound, C_24_H_18_F_2_N_4_OS, contains two crystallographically independent mol­ecules, *A* and *B*, which are linked into two chains of *A* and *B* mol­ecules by inter­molecular N—H⋯O hydrogen bonds. The three-dimensional network is stabilized by π–π inter­actions between the pyridine rings and phenyl rings of different residues, with centroid–centroid distances of 3.793 (1) and 3.666 (2) Å. The mol­ecular conformation is stabilized by intra­molecular N—H⋯F hydrogen bonds (2.15/2.15Å). The imidazole rings make dihedral angles of 39.5 (2)/38.5 (2) and 31.8 (2)/33.2 (2)° with the 4-fluoro­phenyl rings and the pyridine rings, respectively. The methyl group of molecule *A* is disorderd in a 0.60:0.40 ratio.

## Related literature

For related compounds and their biological relevance, see: Ziegler *et al.* (2009 ▶).
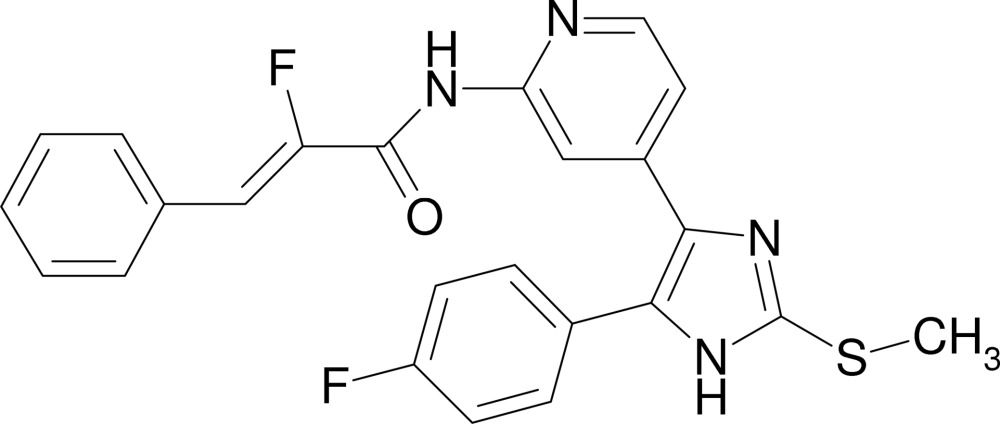


## Experimental

### 

#### Crystal data

C_24_H_18_F_2_N_4_OS
*M* *_r_* = 448.48
Monoclinic, 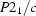

*a* = 20.637 (1) Å
*b* = 7.8413 (5) Å
*c* = 26.256 (2) Å
β = 93.456 (4)°
*V* = 4241.1 (5) Å^3^
*Z* = 8
Mo *K*α radiation
μ = 0.20 mm^−1^
*T* = 173 K
0.49 × 0.41 × 0.03 mm

#### Data collection

Bruker SMART APEXII diffractometer
38383 measured reflections
10251 independent reflections
5719 reflections with *I* > 2σ(*I*)
*R*_int_ = 0.070

#### Refinement

*R*[*F* ^2^ > 2σ(*F* ^2^)] = 0.058
*wR*(*F* ^2^) = 0.149
*S* = 1.02
10251 reflections
589 parameters
H-atom parameters constrained
Δρ_max_ = 0.26 e Å^−3^
Δρ_min_ = −0.40 e Å^−3^

### 

Data collection: *APEX2* (Bruker, 2006 ▶); cell refinement: *SAINT* (Bruker, 2006 ▶); data reduction: *SAINT* program(s) used to solve structure: *SIR97* (Altomare *et al.*, 1999 ▶); program(s) used to refine structure: *SHELXL97* (Sheldrick, 2008 ▶); molecular graphics: *PLATON* (Spek, 2009 ▶); software used to prepare material for publication: *PLATON*.

## Supplementary Material

Crystal structure: contains datablocks I, global. DOI: 10.1107/S1600536809051010/im2165sup1.cif

Structure factors: contains datablocks I. DOI: 10.1107/S1600536809051010/im2165Isup2.hkl

Additional supplementary materials:  crystallographic information; 3D view; checkCIF report

## Figures and Tables

**Table 1:** Hydrogen-bond geometry (Å, °)

*D*—H⋯*A*	*D*—H	H⋯*A*	*D*⋯*A*	*D*—H⋯*A*
N12*A*—H12*A*⋯F9*A*	0.88	2.15	2.605 (2)	112
N21*A*—H21*A*⋯O11*A* ^i^	1.06	1.89	2.813 (2)	143
N12*B*—H12*B*⋯F9*B*	0.88	2.15	2.608 (2)	112
N21*B*—H21*B*⋯O11*B* ^ii^	0.96	1.86	2.796 (2)	165

Symmetry codes: (i) 
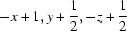
; (ii) 
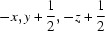
.

**Fig. 1.**
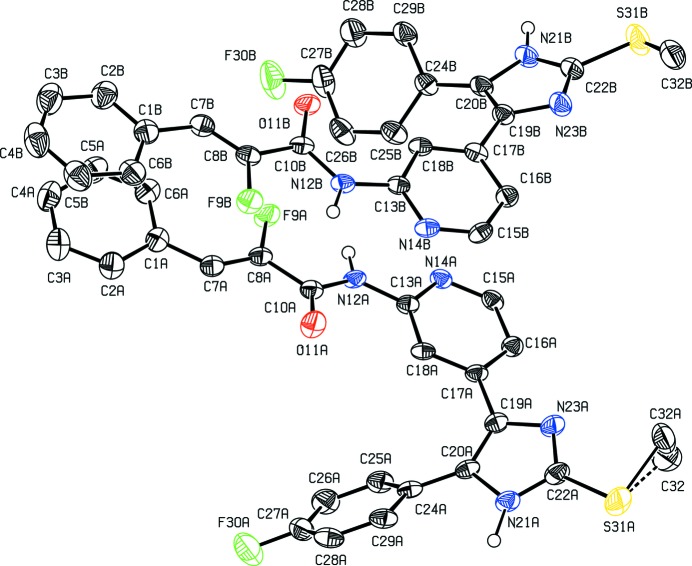
View of compound I. Displacement ellipsoids are drawn at the 50% probability level.

**Fig. 2.**
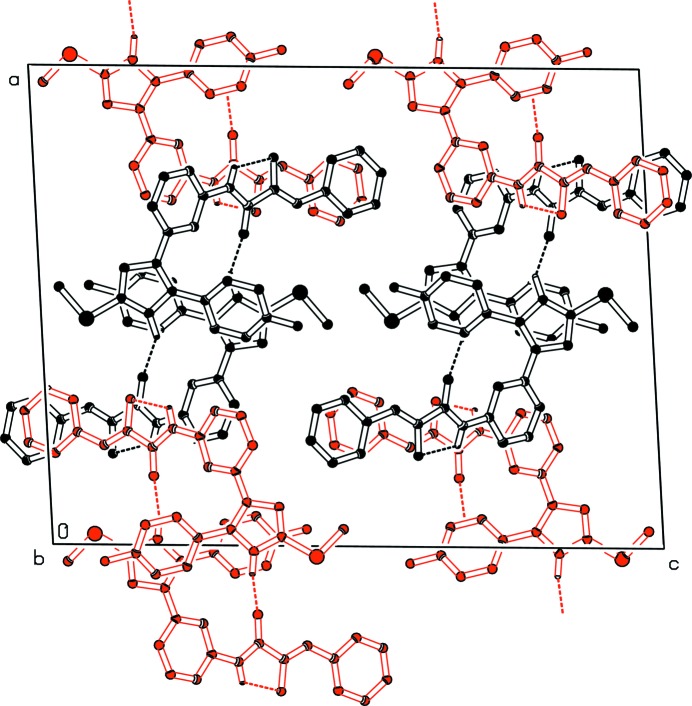
Part of the packing diagram of I. View along the b axis. Hydrogen bonds shown as dashed lines. Molecule A in black, B in red.

## supplementary crystallographic information

### Comment

Inhibition of p38 MAP kinase is a valid approach for the treatment of inflammatory and autoimmune diseases. Imidazoles are preferred scaffolds for p38 MAP kinase inhibition. First generation imidazole based inhibitors like SB202190 or SB203580 suffer from non-mechanistic side effects making more structural modification necessary. Our 2-thioimidazoles proved to have decisive advantages over prototype SB203580-like 2-arylimidazoles and exhibited markedly reduced cytochrome P450 interaction as well as improved pharmacokinetic and metabolic properties. The inhibitor design combines hydrogen bond donors and acceptors to imitate adenine binding, lipophilic moieties adressing a hydrophobic selectivity pocket and a second hydrophobic area. In the crystal structure of the title compound, C_24_H_18_F_2_N_4_OS, the two crystallographically independent molecules (Fig. 1) form a three dimensional network stabilized by π- π interactions between the pyridine rings and phenyl rings with centroid distances of 3.793 (1)Å and 3.666 (2)Å (Fig. 2). The structure also displays intramolecular N—H···F 2.15/2.15Å and intermolecular N—H···O hydrogen bonding 1.89/1.86 Å. The imidazole rings make dihedral angles of 39.5 (2)/38.5 (2)° and 31.8 (2)/33.2 (2)° with the 4-fluorophenyl rings and the pyridine rings, respectively.

### Experimental

1060 mg (2*Z*)-2-fluoro-3-phenylacrylic acid was dissolved in 10 ml *N*-methylpyrrolidinone. After addition of 1032 mg (6.4 mmol) carbonyldiimidazole the mixture was stirred for 20 h at room temperature. 4-[5-(4-fluorophenyl)-2-(methylsulfanyl)-1*H*-imidazole-4-yl]pyridine-2-amine was added and the reaction mixture was heated to 120°C for 4 h. The reaction mixture was quenched with a solution of concentrated sodium hydrogen carbonate and extracted with ethyl acetate. The crude product was purified by flash chromatography (Eluent: ethyl acetate/petroleum ether 1/1) to yield 460 mg (52%) of the title compound. Crystals suitable for X-ray analysis were obtained by slow crystallization from ethyl acetate/petroleum ether.

### Refinement

Hydrogen atoms attached to carbons were placed at calculated positions with C—H = 0.95 Å (aromatic) or 0.98–0.99 Å (*sp*^3^ C-atom). Hydrogen atoms attached to nitrogen were located from diff. Fourier maps. All H atoms were refined in the riding-model approximation with isotropic displacement parameters (set at 1.2–1.5 times the *U*_eq_ of the parent atom). The methyl group of molecule A is disorderd (s.o.f.=0.4/0.6).

### Crystal data

**Table:**

C_24_H_18_F_2_N_4_OS	*F*(000) = 1856
*M**_r_* = 448.48	*D*_x_ = 1.405 Mg m^−^^3^
Monoclinic, *P*2_1_/*c*	Mo *K*α radiation, λ = 0.71069 Å
Hall symbol: -P 2ybc	Cell parameters from 6588 reflections
*a* = 20.637 (1) Å	θ = 2.4–24.5°
*b* = 7.8413 (5) Å	µ = 0.20 mm^−^^1^
*c* = 26.256 (2) Å	*T* = 173 K
β = 93.456 (4)°	Plate, colourless
*V* = 4241.1 (5) Å^3^	0.49 × 0.41 × 0.03 mm
*Z* = 8	

### Data collection

**Table:**

Bruker SMART APEXII diffractometer	5719 reflections with *I* > 2σ(*I*)
Radiation source: sealed Tube	*R*_int_ = 0.070
graphite	θ_max_ = 28.0°, θ_min_ = 1.8°
CCD scan	*h* = −27→27
38383 measured reflections	*k* = −10→10
10251 independent reflections	*l* = −34→34

### Refinement

**Table:**

Refinement on *F*^2^	Primary atom site location: structure-invariant direct methods
Least-squares matrix: full	Secondary atom site location: difference Fourier map
*R*[*F*^2^ > 2σ(*F*^2^)] = 0.058	Hydrogen site location: inferred from neighbouring sites
*wR*(*F*^2^) = 0.149	H-atom parameters constrained
*S* = 1.02	*w* = 1/[σ^2^(*F*_o_^2^) + (0.0647*P*)^2^ + 0.5112*P*] where *P* = (*F*_o_^2^ + 2*F*_c_^2^)/3
10251 reflections	(Δ/σ)_max_ < 0.001
589 parameters	Δρ_max_ = 0.26 e Å^−^^3^
0 restraints	Δρ_min_ = −0.40 e Å^−^^3^

### Special details

**Table:**

Geometry. All e.s.d.'s (except the e.s.d. in the dihedral angle between two l.s. planes) are estimated using the full covariance matrix. The cell e.s.d.'s are taken into account individually in the estimation of e.s.d.'s in distances, angles and torsion angles; correlations between e.s.d.'s in cell parameters are only used when they are defined by crystal symmetry. An approximate (isotropic) treatment of cell e.s.d.'s is used for estimating e.s.d.'s involving l.s. planes.
Refinement. Refinement of *F*^2^ against ALL reflections. The weighted *R*-factor *wR* and goodness of fit *S* are based on *F*^2^, conventional *R*-factors *R* are based on *F*, with *F* set to zero for negative *F*^2^. The threshold expression of *F*^2^ > σ(*F*^2^) is used only for calculating *R*-factors(gt) *etc*. and is not relevant to the choice of reflections for refinement. *R*-factors based on *F*^2^ are statistically about twice as large as those based on *F*, and *R*- factors based on ALL data will be even larger.

### Fractional atomic coordinates and isotropic or equivalent isotropic displacement parameters (Å^2^)

**Table:**

	*x*	*y*	*z*	*U*_iso_*/*U*_eq_	Occ. (<1)
C1A	0.25992 (11)	0.0543 (3)	0.02402 (9)	0.0321 (6)	
C2A	0.30501 (12)	0.0017 (4)	−0.01049 (9)	0.0403 (7)	
H2A	0.3500	0.0170	−0.0020	0.048*	
C3A	0.28548 (14)	−0.0717 (4)	−0.05651 (10)	0.0479 (8)	
H3A	0.3168	−0.1059	−0.0796	0.057*	
C4A	0.22002 (14)	−0.0955 (4)	−0.06890 (10)	0.0464 (7)	
H4A	0.2062	−0.1471	−0.1005	0.056*	
C5A	0.17455 (13)	−0.0441 (4)	−0.03539 (10)	0.0446 (7)	
H5A	0.1297	−0.0604	−0.0442	0.054*	
C6A	0.19366 (12)	0.0305 (3)	0.01078 (9)	0.0361 (6)	
H6A	0.1620	0.0656	0.0335	0.043*	
C7A	0.28528 (11)	0.1331 (3)	0.07146 (9)	0.0312 (6)	
H7A	0.3310	0.1244	0.0774	0.037*	
C8A	0.25600 (10)	0.2143 (3)	0.10751 (9)	0.0286 (6)	
F9A	0.19015 (6)	0.23695 (19)	0.10585 (5)	0.0387 (4)	
C10A	0.29076 (10)	0.2946 (3)	0.15220 (9)	0.0278 (6)	
O11A	0.34927 (7)	0.2716 (3)	0.16114 (7)	0.0431 (5)	
N12A	0.25345 (8)	0.3933 (3)	0.18095 (7)	0.0270 (5)	
H12A	0.2120	0.3966	0.1710	0.032*	
C13A	0.27134 (10)	0.4916 (3)	0.22450 (8)	0.0255 (5)	
N14A	0.21954 (8)	0.5586 (3)	0.24543 (7)	0.0274 (5)	
C15A	0.23191 (10)	0.6546 (3)	0.28706 (8)	0.0277 (6)	
H15A	0.1960	0.7026	0.3030	0.033*	
C16A	0.29363 (10)	0.6884 (3)	0.30840 (9)	0.0278 (6)	
H16A	0.2995	0.7569	0.3382	0.033*	
C17A	0.34717 (10)	0.6201 (3)	0.28535 (8)	0.0261 (5)	
C18A	0.33531 (10)	0.5178 (3)	0.24258 (9)	0.0273 (6)	
H18A	0.3701	0.4668	0.2261	0.033*	
C19A	0.41346 (11)	0.6550 (3)	0.30710 (9)	0.0306 (6)	
C20A	0.47089 (10)	0.6726 (3)	0.28335 (9)	0.0289 (6)	
N21A	0.51724 (9)	0.7024 (3)	0.32321 (7)	0.0327 (5)	
H21A	0.5655	0.7248	0.3126	0.039*	
C22A	0.48613 (11)	0.7018 (4)	0.36716 (9)	0.0349 (6)	
N23A	0.42318 (9)	0.6751 (3)	0.35963 (7)	0.0345 (5)	
C24A	0.48868 (10)	0.6741 (3)	0.23022 (9)	0.0295 (6)	
C25A	0.44859 (11)	0.7483 (4)	0.19170 (9)	0.0355 (6)	
H25A	0.4085	0.7971	0.2003	0.043*	
C26A	0.46526 (12)	0.7532 (4)	0.14130 (10)	0.0429 (7)	
H26A	0.4376	0.8049	0.1155	0.051*	
C27A	0.52376 (12)	0.6797 (4)	0.13016 (10)	0.0405 (7)	
C28A	0.56466 (11)	0.6057 (4)	0.16634 (10)	0.0395 (7)	
H28A	0.6043	0.5556	0.1572	0.047*	
C29A	0.54766 (10)	0.6045 (4)	0.21661 (10)	0.0353 (6)	
H29A	0.5765	0.5557	0.2422	0.042*	
F30A	0.54117 (8)	0.6843 (3)	0.08099 (6)	0.0651 (5)	
S31A	0.52898 (3)	0.73578 (12)	0.42603 (3)	0.0509 (2)	
C32	0.4655 (10)	0.760 (2)	0.4639 (8)	0.066 (6)	0.40
H32A	0.4338	0.8390	0.4476	0.099*	0.40
H32B	0.4813	0.8060	0.4971	0.099*	0.40
H32C	0.4449	0.6492	0.4688	0.099*	0.40
C32A	0.4635 (7)	0.6773 (15)	0.4686 (5)	0.062 (3)	0.60
H321	0.4268	0.7556	0.4628	0.093*	0.60
H322	0.4801	0.6852	0.5043	0.093*	0.60
H323	0.4491	0.5603	0.4612	0.093*	0.60
C1B	0.23464 (12)	−0.3888 (3)	0.05008 (9)	0.0360 (6)	
C2B	0.19075 (14)	−0.4497 (4)	0.01153 (10)	0.0454 (7)	
H2B	0.1456	−0.4324	0.0145	0.054*	
C3B	0.21166 (16)	−0.5348 (4)	−0.03086 (11)	0.0543 (8)	
H3B	0.1812	−0.5749	−0.0567	0.065*	
C4B	0.27734 (17)	−0.5600 (4)	−0.03490 (12)	0.0579 (9)	
H4B	0.2923	−0.6166	−0.0640	0.069*	
C5B	0.32139 (15)	−0.5038 (4)	0.00296 (11)	0.0515 (8)	
H5B	0.3664	−0.5241	0.0000	0.062*	
C6B	0.30084 (13)	−0.4178 (4)	0.04540 (10)	0.0420 (7)	
H6B	0.3317	−0.3788	0.0711	0.050*	
C7B	0.20795 (12)	−0.2954 (3)	0.09253 (9)	0.0323 (6)	
H7B	0.1619	−0.2916	0.0915	0.039*	
C8B	0.23724 (10)	−0.2157 (3)	0.13195 (9)	0.0291 (6)	
F9B	0.30350 (6)	−0.2114 (2)	0.13905 (5)	0.0390 (4)	
C10B	0.20370 (10)	−0.1218 (3)	0.17144 (9)	0.0276 (6)	
O11B	0.14409 (7)	−0.1189 (2)	0.17073 (6)	0.0390 (5)	
N12B	0.24300 (8)	−0.0391 (3)	0.20664 (7)	0.0276 (5)	
H12B	0.2848	−0.0462	0.2019	0.033*	
C13B	0.22721 (10)	0.0563 (3)	0.24973 (8)	0.0254 (5)	
N14B	0.28014 (8)	0.1086 (3)	0.27719 (7)	0.0298 (5)	
C15B	0.26994 (11)	0.1989 (3)	0.31897 (9)	0.0310 (6)	
H15B	0.3068	0.2383	0.3390	0.037*	
C16B	0.20928 (11)	0.2391 (3)	0.33507 (9)	0.0301 (6)	
H16B	0.2050	0.3007	0.3658	0.036*	
C17B	0.15426 (10)	0.1873 (3)	0.30525 (9)	0.0276 (6)	
C18B	0.16364 (10)	0.0927 (3)	0.26147 (9)	0.0266 (6)	
H18B	0.1278	0.0540	0.2402	0.032*	
C19B	0.08965 (11)	0.2330 (3)	0.32120 (9)	0.0287 (6)	
C20B	0.03289 (10)	0.2665 (3)	0.29255 (9)	0.0292 (6)	
N21B	−0.01221 (9)	0.3001 (3)	0.32831 (7)	0.0350 (5)	
H21B	−0.0585	0.3148	0.3238	0.042*	
C22B	0.01860 (11)	0.2882 (4)	0.37505 (10)	0.0365 (6)	
N23B	0.08067 (9)	0.2491 (3)	0.37314 (8)	0.0356 (5)	
C24B	0.01542 (10)	0.2784 (3)	0.23753 (9)	0.0300 (6)	
C25B	0.05810 (11)	0.3511 (4)	0.20433 (9)	0.0363 (7)	
H25B	0.0991	0.3916	0.2175	0.044*	
C26B	0.04173 (12)	0.3652 (4)	0.15269 (10)	0.0428 (7)	
H26B	0.0709	0.4152	0.1304	0.051*	
C27B	−0.01775 (13)	0.3051 (4)	0.13445 (9)	0.0458 (8)	
C28B	−0.06105 (13)	0.2328 (4)	0.16528 (10)	0.0493 (8)	
H28B	−0.1017	0.1918	0.1514	0.059*	
C29B	−0.04481 (11)	0.2201 (4)	0.21709 (9)	0.0386 (7)	
H29B	−0.0748	0.1714	0.2390	0.046*	
F30B	−0.03488 (8)	0.3204 (3)	0.08353 (6)	0.0688 (6)	
S31B	−0.02231 (3)	0.32522 (12)	0.43068 (3)	0.0545 (2)	
C32B	0.03538 (14)	0.2332 (5)	0.47673 (10)	0.0579 (9)	
H32D	0.0357	0.1090	0.4726	0.087*	
H32E	0.0232	0.2618	0.5112	0.087*	
H32F	0.0787	0.2785	0.4715	0.087*	

### Atomic displacement parameters (Å^2^)

**Table:**

	*U*^11^	*U*^22^	*U*^33^	*U*^12^	*U*^13^	*U*^23^
C1A	0.0375 (14)	0.0252 (15)	0.0331 (13)	0.0026 (12)	−0.0016 (10)	0.0026 (12)
C2A	0.0358 (14)	0.0448 (19)	0.0402 (15)	0.0058 (13)	0.0005 (11)	−0.0030 (14)
C3A	0.0564 (18)	0.050 (2)	0.0372 (15)	0.0090 (15)	0.0038 (13)	−0.0065 (15)
C4A	0.0573 (18)	0.046 (2)	0.0347 (15)	−0.0010 (15)	−0.0082 (13)	−0.0050 (14)
C5A	0.0417 (16)	0.044 (2)	0.0472 (16)	−0.0011 (14)	−0.0084 (13)	−0.0013 (15)
C6A	0.0384 (14)	0.0327 (17)	0.0367 (14)	0.0000 (12)	−0.0024 (11)	0.0026 (13)
C7A	0.0273 (12)	0.0304 (16)	0.0356 (13)	−0.0002 (11)	−0.0001 (10)	0.0022 (12)
C8A	0.0189 (11)	0.0308 (15)	0.0359 (13)	−0.0020 (10)	0.0000 (9)	0.0018 (12)
F9A	0.0204 (7)	0.0498 (10)	0.0454 (8)	−0.0009 (6)	−0.0031 (6)	−0.0100 (8)
C10A	0.0219 (12)	0.0297 (16)	0.0317 (12)	−0.0021 (10)	0.0011 (9)	0.0042 (12)
O11A	0.0171 (8)	0.0648 (15)	0.0467 (10)	0.0069 (8)	−0.0036 (7)	−0.0150 (10)
N12A	0.0139 (9)	0.0315 (13)	0.0354 (11)	−0.0016 (8)	−0.0008 (8)	−0.0028 (10)
C13A	0.0194 (11)	0.0257 (15)	0.0311 (12)	0.0002 (10)	0.0001 (9)	0.0036 (11)
N14A	0.0188 (9)	0.0333 (13)	0.0301 (10)	−0.0005 (9)	0.0016 (8)	0.0024 (10)
C15A	0.0198 (11)	0.0339 (16)	0.0298 (12)	0.0018 (10)	0.0044 (9)	0.0027 (12)
C16A	0.0262 (12)	0.0296 (15)	0.0275 (12)	−0.0013 (11)	0.0001 (9)	0.0014 (12)
C17A	0.0185 (11)	0.0294 (15)	0.0301 (12)	−0.0032 (10)	−0.0018 (9)	0.0036 (11)
C18A	0.0151 (11)	0.0301 (15)	0.0368 (13)	−0.0020 (10)	0.0017 (9)	0.0011 (12)
C19A	0.0230 (12)	0.0324 (16)	0.0359 (13)	−0.0019 (11)	−0.0027 (10)	0.0001 (12)
C20A	0.0195 (11)	0.0295 (15)	0.0368 (13)	−0.0020 (10)	−0.0054 (9)	−0.0004 (12)
N21A	0.0208 (10)	0.0396 (14)	0.0371 (12)	−0.0025 (9)	−0.0044 (8)	−0.0007 (11)
C22A	0.0248 (12)	0.0414 (18)	0.0374 (14)	−0.0025 (12)	−0.0074 (10)	−0.0029 (13)
N23A	0.0232 (10)	0.0424 (15)	0.0369 (12)	−0.0024 (9)	−0.0058 (8)	−0.0024 (11)
C24A	0.0188 (11)	0.0304 (16)	0.0390 (14)	−0.0060 (10)	−0.0022 (9)	0.0009 (12)
C25A	0.0214 (12)	0.0422 (18)	0.0424 (15)	−0.0001 (11)	−0.0032 (10)	0.0026 (13)
C26A	0.0309 (14)	0.055 (2)	0.0416 (15)	−0.0021 (13)	−0.0058 (11)	0.0048 (14)
C27A	0.0330 (14)	0.053 (2)	0.0354 (14)	−0.0089 (13)	0.0035 (11)	0.0034 (14)
C28A	0.0214 (12)	0.0431 (18)	0.0545 (17)	−0.0028 (12)	0.0049 (11)	0.0032 (15)
C29A	0.0190 (11)	0.0404 (17)	0.0460 (15)	−0.0027 (11)	−0.0029 (10)	0.0069 (14)
F30A	0.0505 (10)	0.1027 (16)	0.0431 (9)	−0.0009 (10)	0.0123 (8)	0.0097 (10)
S31A	0.0308 (4)	0.0783 (7)	0.0419 (4)	−0.0027 (4)	−0.0127 (3)	−0.0081 (4)
C32	0.046 (6)	0.114 (17)	0.035 (6)	−0.021 (10)	−0.012 (4)	0.009 (9)
C32A	0.040 (4)	0.110 (10)	0.037 (4)	−0.006 (6)	0.007 (3)	−0.009 (6)
C1B	0.0474 (16)	0.0239 (15)	0.0372 (14)	−0.0009 (12)	0.0056 (12)	0.0035 (13)
C2B	0.0563 (18)	0.0349 (18)	0.0446 (16)	−0.0010 (14)	0.0008 (13)	−0.0017 (15)
C3B	0.079 (2)	0.040 (2)	0.0437 (17)	−0.0031 (17)	0.0020 (15)	−0.0050 (16)
C4B	0.097 (3)	0.033 (2)	0.0457 (18)	0.0049 (18)	0.0202 (18)	−0.0004 (16)
C5B	0.063 (2)	0.040 (2)	0.0534 (18)	0.0052 (16)	0.0195 (15)	−0.0011 (16)
C6B	0.0485 (16)	0.0308 (17)	0.0476 (16)	0.0003 (13)	0.0105 (13)	0.0003 (14)
C7B	0.0297 (13)	0.0296 (16)	0.0377 (14)	0.0001 (11)	0.0040 (10)	0.0034 (13)
C8B	0.0204 (11)	0.0303 (16)	0.0366 (13)	0.0019 (10)	0.0015 (10)	0.0055 (12)
F9B	0.0201 (7)	0.0496 (11)	0.0472 (9)	0.0060 (6)	0.0011 (6)	−0.0059 (8)
C10B	0.0206 (12)	0.0268 (15)	0.0353 (13)	−0.0021 (10)	0.0020 (9)	0.0062 (12)
O11B	0.0178 (8)	0.0516 (13)	0.0475 (10)	−0.0040 (8)	0.0013 (7)	−0.0078 (9)
N12B	0.0143 (9)	0.0303 (13)	0.0385 (11)	0.0018 (8)	0.0035 (8)	−0.0001 (10)
C13B	0.0191 (11)	0.0246 (15)	0.0327 (13)	0.0002 (10)	0.0028 (9)	0.0029 (11)
N14B	0.0177 (9)	0.0328 (13)	0.0387 (11)	−0.0014 (9)	−0.0003 (8)	0.0007 (11)
C15B	0.0223 (12)	0.0328 (16)	0.0370 (14)	−0.0021 (11)	−0.0062 (10)	−0.0019 (13)
C16B	0.0260 (12)	0.0336 (16)	0.0306 (12)	−0.0001 (11)	0.0007 (9)	0.0007 (12)
C17B	0.0214 (11)	0.0302 (15)	0.0314 (12)	−0.0006 (10)	0.0034 (9)	0.0063 (12)
C18B	0.0148 (10)	0.0294 (15)	0.0357 (13)	0.0010 (10)	0.0011 (9)	0.0017 (12)
C19B	0.0231 (12)	0.0321 (16)	0.0312 (13)	0.0007 (11)	0.0042 (9)	−0.0001 (12)
C20B	0.0198 (11)	0.0330 (16)	0.0355 (13)	0.0013 (10)	0.0080 (9)	−0.0010 (12)
N21B	0.0211 (10)	0.0463 (15)	0.0382 (12)	0.0016 (10)	0.0075 (8)	−0.0024 (11)
C22B	0.0275 (13)	0.0459 (19)	0.0367 (14)	−0.0031 (12)	0.0076 (11)	−0.0034 (13)
N23B	0.0272 (11)	0.0460 (15)	0.0342 (11)	0.0027 (10)	0.0066 (8)	−0.0037 (11)
C24B	0.0199 (11)	0.0356 (17)	0.0350 (13)	0.0073 (11)	0.0056 (9)	0.0020 (12)
C25B	0.0237 (12)	0.0422 (18)	0.0436 (15)	0.0051 (12)	0.0066 (10)	0.0055 (14)
C26B	0.0349 (14)	0.054 (2)	0.0408 (15)	0.0085 (13)	0.0133 (12)	0.0053 (14)
C27B	0.0374 (15)	0.071 (2)	0.0289 (14)	0.0147 (15)	0.0028 (11)	−0.0002 (15)
C28B	0.0281 (14)	0.078 (2)	0.0412 (15)	0.0041 (14)	0.0009 (11)	−0.0001 (16)
C29B	0.0231 (12)	0.057 (2)	0.0366 (14)	0.0035 (12)	0.0059 (10)	−0.0005 (14)
F30B	0.0563 (11)	0.1181 (18)	0.0319 (9)	0.0141 (11)	0.0019 (7)	0.0054 (10)
S31B	0.0370 (4)	0.0870 (7)	0.0411 (4)	0.0055 (4)	0.0154 (3)	−0.0076 (4)
C32B	0.0510 (18)	0.087 (3)	0.0360 (15)	−0.0105 (17)	0.0088 (13)	0.0013 (17)

### Geometric parameters (Å, °)

**Table:**

C1A—C2A	1.400 (3)	C32A—H322	0.9800
C1A—C6A	1.403 (3)	C32A—H323	0.9800
C1A—C7A	1.459 (3)	C1B—C6B	1.398 (4)
C2A—C3A	1.377 (4)	C1B—C2B	1.401 (4)
C2A—H2A	0.9500	C1B—C7B	1.468 (3)
C3A—C4A	1.383 (4)	C2B—C3B	1.389 (4)
C3A—H3A	0.9500	C2B—H2B	0.9500
C4A—C5A	1.384 (4)	C3B—C4B	1.380 (4)
C4A—H4A	0.9500	C3B—H3B	0.9500
C5A—C6A	1.382 (3)	C4B—C5B	1.379 (4)
C5A—H5A	0.9500	C4B—H4B	0.9500
C6A—H6A	0.9500	C5B—C6B	1.391 (4)
C7A—C8A	1.317 (3)	C5B—H5B	0.9500
C7A—H7A	0.9500	C6B—H6B	0.9500
C8A—F9A	1.369 (2)	C7B—C8B	1.324 (3)
C8A—C10A	1.479 (3)	C7B—H7B	0.9500
C10A—O11A	1.230 (3)	C8B—F9B	1.369 (2)
C10A—N12A	1.353 (3)	C8B—C10B	1.478 (3)
N12A—C13A	1.410 (3)	C10B—O11B	1.229 (2)
N12A—H12A	0.8800	C10B—N12B	1.357 (3)
C13A—N14A	1.338 (3)	N12B—C13B	1.411 (3)
C13A—C18A	1.391 (3)	N12B—H12B	0.8800
N14A—C15A	1.339 (3)	C13B—N14B	1.336 (3)
C15A—C16A	1.386 (3)	C13B—C18B	1.395 (3)
C15A—H15A	0.9500	N14B—C15B	1.333 (3)
C16A—C17A	1.398 (3)	C15B—C16B	1.382 (3)
C16A—H16A	0.9500	C15B—H15B	0.9500
C17A—C18A	1.389 (3)	C16B—C17B	1.400 (3)
C17A—C19A	1.476 (3)	C16B—H16B	0.9500
C18A—H18A	0.9500	C17B—C18B	1.392 (3)
C19A—C20A	1.379 (3)	C17B—C19B	1.466 (3)
C19A—N23A	1.391 (3)	C18B—H18B	0.9500
C20A—N21A	1.395 (3)	C19B—C20B	1.379 (3)
C20A—C24A	1.464 (3)	C19B—N23B	1.393 (3)
N21A—C22A	1.354 (3)	C20B—N21B	1.387 (3)
N21A—H21A	1.0641	C20B—C24B	1.470 (3)
C22A—N23A	1.319 (3)	N21B—C22B	1.351 (3)
C22A—S31A	1.754 (2)	N21B—H21B	0.9614
C24A—C25A	1.395 (3)	C22B—N23B	1.321 (3)
C24A—C29A	1.400 (3)	C22B—S31B	1.755 (2)
C25A—C26A	1.388 (3)	C24B—C25B	1.398 (3)
C25A—H25A	0.9500	C24B—C29B	1.400 (3)
C26A—C27A	1.385 (4)	C25B—C26B	1.382 (3)
C26A—H26A	0.9500	C25B—H25B	0.9500
C27A—F30A	1.361 (3)	C26B—C27B	1.374 (4)
C27A—C28A	1.362 (4)	C26B—H26B	0.9500
C28A—C29A	1.386 (3)	C27B—C28B	1.365 (4)
C28A—H28A	0.9500	C27B—F30B	1.367 (3)
C29A—H29A	0.9500	C28B—C29B	1.385 (3)
S31A—C32	1.70 (2)	C28B—H28B	0.9500
S31A—C32A	1.863 (12)	C29B—H29B	0.9500
C32—H32A	0.9800	S31B—C32B	1.796 (3)
C32—H32B	0.9800	C32B—H32D	0.9800
C32—H32C	0.9800	C32B—H32E	0.9800
C32A—H321	0.9800	C32B—H32F	0.9800
			
C2A—C1A—C6A	118.5 (2)	H321—C32A—H323	109.5
C2A—C1A—C7A	117.3 (2)	H322—C32A—H323	109.5
C6A—C1A—C7A	124.2 (2)	C6B—C1B—C2B	118.3 (2)
C3A—C2A—C1A	121.4 (2)	C6B—C1B—C7B	124.1 (2)
C3A—C2A—H2A	119.3	C2B—C1B—C7B	117.6 (2)
C1A—C2A—H2A	119.3	C3B—C2B—C1B	121.6 (3)
C2A—C3A—C4A	119.5 (3)	C3B—C2B—H2B	119.2
C2A—C3A—H3A	120.2	C1B—C2B—H2B	119.2
C4A—C3A—H3A	120.2	C4B—C3B—C2B	119.0 (3)
C3A—C4A—C5A	120.1 (3)	C4B—C3B—H3B	120.5
C3A—C4A—H4A	120.0	C2B—C3B—H3B	120.5
C5A—C4A—H4A	120.0	C5B—C4B—C3B	120.4 (3)
C6A—C5A—C4A	120.8 (2)	C5B—C4B—H4B	119.8
C6A—C5A—H5A	119.6	C3B—C4B—H4B	119.8
C4A—C5A—H5A	119.6	C4B—C5B—C6B	120.9 (3)
C5A—C6A—C1A	119.7 (2)	C4B—C5B—H5B	119.6
C5A—C6A—H6A	120.1	C6B—C5B—H5B	119.6
C1A—C6A—H6A	120.1	C5B—C6B—C1B	119.8 (3)
C8A—C7A—C1A	131.4 (2)	C5B—C6B—H6B	120.1
C8A—C7A—H7A	114.3	C1B—C6B—H6B	120.1
C1A—C7A—H7A	114.3	C8B—C7B—C1B	130.9 (2)
C7A—C8A—F9A	122.5 (2)	C8B—C7B—H7B	114.6
C7A—C8A—C10A	123.7 (2)	C1B—C7B—H7B	114.6
F9A—C8A—C10A	113.77 (19)	C7B—C8B—F9B	121.6 (2)
O11A—C10A—N12A	124.2 (2)	C7B—C8B—C10B	125.0 (2)
O11A—C10A—C8A	120.8 (2)	F9B—C8B—C10B	113.5 (2)
N12A—C10A—C8A	114.91 (19)	O11B—C10B—N12B	123.9 (2)
C10A—N12A—C13A	129.43 (18)	O11B—C10B—C8B	120.6 (2)
C10A—N12A—H12A	115.3	N12B—C10B—C8B	115.46 (19)
C13A—N12A—H12A	115.3	C10B—N12B—C13B	129.94 (18)
N14A—C13A—C18A	124.6 (2)	C10B—N12B—H12B	115.0
N14A—C13A—N12A	111.76 (18)	C13B—N12B—H12B	115.0
C18A—C13A—N12A	123.7 (2)	N14B—C13B—C18B	124.5 (2)
C13A—N14A—C15A	115.97 (18)	N14B—C13B—N12B	112.00 (18)
N14A—C15A—C16A	124.3 (2)	C18B—C13B—N12B	123.5 (2)
N14A—C15A—H15A	117.9	C15B—N14B—C13B	116.26 (19)
C16A—C15A—H15A	117.9	N14B—C15B—C16B	124.3 (2)
C15A—C16A—C17A	118.9 (2)	N14B—C15B—H15B	117.8
C15A—C16A—H16A	120.6	C16B—C15B—H15B	117.8
C17A—C16A—H16A	120.6	C15B—C16B—C17B	118.8 (2)
C18A—C17A—C16A	117.77 (19)	C15B—C16B—H16B	120.6
C18A—C17A—C19A	122.3 (2)	C17B—C16B—H16B	120.6
C16A—C17A—C19A	120.0 (2)	C18B—C17B—C16B	117.9 (2)
C17A—C18A—C13A	118.6 (2)	C18B—C17B—C19B	122.7 (2)
C17A—C18A—H18A	120.7	C16B—C17B—C19B	119.4 (2)
C13A—C18A—H18A	120.7	C17B—C18B—C13B	118.1 (2)
C20A—C19A—N23A	111.14 (19)	C17B—C18B—H18B	120.9
C20A—C19A—C17A	130.2 (2)	C13B—C18B—H18B	120.9
N23A—C19A—C17A	118.68 (19)	C20B—C19B—N23B	111.0 (2)
C19A—C20A—N21A	104.3 (2)	C20B—C19B—C17B	130.4 (2)
C19A—C20A—C24A	134.7 (2)	N23B—C19B—C17B	118.6 (2)
N21A—C20A—C24A	120.91 (19)	C19B—C20B—N21B	104.5 (2)
C22A—N21A—C20A	107.46 (19)	C19B—C20B—C24B	134.3 (2)
C22A—N21A—H21A	136.3	N21B—C20B—C24B	121.2 (2)
C20A—N21A—H21A	116.2	C22B—N21B—C20B	107.68 (19)
N23A—C22A—N21A	112.7 (2)	C22B—N21B—H21B	122.0
N23A—C22A—S31A	126.6 (2)	C20B—N21B—H21B	129.9
N21A—C22A—S31A	120.71 (17)	N23B—C22B—N21B	112.7 (2)
C22A—N23A—C19A	104.40 (19)	N23B—C22B—S31B	125.9 (2)
C25A—C24A—C29A	117.7 (2)	N21B—C22B—S31B	121.39 (18)
C25A—C24A—C20A	121.5 (2)	C22B—N23B—C19B	104.2 (2)
C29A—C24A—C20A	120.8 (2)	C25B—C24B—C29B	118.2 (2)
C26A—C25A—C24A	122.2 (2)	C25B—C24B—C20B	120.8 (2)
C26A—C25A—H25A	118.9	C29B—C24B—C20B	121.0 (2)
C24A—C25A—H25A	118.9	C26B—C25B—C24B	121.2 (2)
C27A—C26A—C25A	117.3 (2)	C26B—C25B—H25B	119.4
C27A—C26A—H26A	121.3	C24B—C25B—H25B	119.4
C25A—C26A—H26A	121.3	C27B—C26B—C25B	118.4 (2)
F30A—C27A—C28A	119.0 (2)	C27B—C26B—H26B	120.8
F30A—C27A—C26A	118.2 (2)	C25B—C26B—H26B	120.8
C28A—C27A—C26A	122.8 (2)	C28B—C27B—F30B	118.5 (2)
C27A—C28A—C29A	119.0 (2)	C28B—C27B—C26B	122.6 (2)
C27A—C28A—H28A	120.5	F30B—C27B—C26B	118.9 (2)
C29A—C28A—H28A	120.5	C27B—C28B—C29B	118.9 (3)
C28A—C29A—C24A	120.9 (2)	C27B—C28B—H28B	120.5
C28A—C29A—H29A	119.5	C29B—C28B—H28B	120.5
C24A—C29A—H29A	119.5	C28B—C29B—C24B	120.7 (2)
C32—S31A—C22A	99.6 (7)	C28B—C29B—H29B	119.7
C22A—S31A—C32A	98.4 (5)	C24B—C29B—H29B	119.7
S31A—C32—H32A	109.5	C22B—S31B—C32B	99.27 (13)
S31A—C32—H32B	109.5	S31B—C32B—H32D	109.5
S31A—C32—H32C	109.5	S31B—C32B—H32E	109.5
S31A—C32A—H321	109.5	H32D—C32B—H32E	109.5
S31A—C32A—H322	109.5	S31B—C32B—H32F	109.5
H321—C32A—H322	109.5	H32D—C32B—H32F	109.5
S31A—C32A—H323	109.5	H32E—C32B—H32F	109.5
			
C6A—C1A—C2A—C3A	0.0 (4)	N21A—C22A—S31A—C32A	−169.5 (5)
C7A—C1A—C2A—C3A	−179.1 (3)	C6B—C1B—C2B—C3B	−0.9 (4)
C1A—C2A—C3A—C4A	−0.5 (4)	C7B—C1B—C2B—C3B	178.0 (3)
C2A—C3A—C4A—C5A	0.6 (5)	C1B—C2B—C3B—C4B	0.2 (4)
C3A—C4A—C5A—C6A	−0.3 (5)	C2B—C3B—C4B—C5B	0.9 (5)
C4A—C5A—C6A—C1A	−0.2 (4)	C3B—C4B—C5B—C6B	−1.3 (5)
C2A—C1A—C6A—C5A	0.3 (4)	C4B—C5B—C6B—C1B	0.5 (4)
C7A—C1A—C6A—C5A	179.4 (3)	C2B—C1B—C6B—C5B	0.6 (4)
C2A—C1A—C7A—C8A	169.8 (3)	C7B—C1B—C6B—C5B	−178.3 (3)
C6A—C1A—C7A—C8A	−9.3 (4)	C6B—C1B—C7B—C8B	3.5 (4)
C1A—C7A—C8A—F9A	1.0 (4)	C2B—C1B—C7B—C8B	−175.4 (3)
C1A—C7A—C8A—C10A	−176.6 (3)	C1B—C7B—C8B—F9B	−0.7 (4)
C7A—C8A—C10A—O11A	−8.9 (4)	C1B—C7B—C8B—C10B	178.1 (2)
F9A—C8A—C10A—O11A	173.3 (2)	C7B—C8B—C10B—O11B	3.2 (4)
C7A—C8A—C10A—N12A	170.6 (2)	F9B—C8B—C10B—O11B	−177.9 (2)
F9A—C8A—C10A—N12A	−7.2 (3)	C7B—C8B—C10B—N12B	−175.6 (2)
O11A—C10A—N12A—C13A	1.9 (4)	F9B—C8B—C10B—N12B	3.3 (3)
C8A—C10A—N12A—C13A	−177.6 (2)	O11B—C10B—N12B—C13B	3.6 (4)
C10A—N12A—C13A—N14A	−173.8 (2)	C8B—C10B—N12B—C13B	−177.7 (2)
C10A—N12A—C13A—C18A	7.4 (4)	C10B—N12B—C13B—N14B	175.7 (2)
C18A—C13A—N14A—C15A	−1.0 (3)	C10B—N12B—C13B—C18B	−5.0 (4)
N12A—C13A—N14A—C15A	−179.9 (2)	C18B—C13B—N14B—C15B	1.2 (4)
C13A—N14A—C15A—C16A	0.8 (3)	N12B—C13B—N14B—C15B	−179.5 (2)
N14A—C15A—C16A—C17A	0.5 (4)	C13B—N14B—C15B—C16B	0.5 (4)
C15A—C16A—C17A—C18A	−1.4 (3)	N14B—C15B—C16B—C17B	−2.1 (4)
C15A—C16A—C17A—C19A	179.6 (2)	C15B—C16B—C17B—C18B	2.0 (4)
C16A—C17A—C18A—C13A	1.2 (3)	C15B—C16B—C17B—C19B	−178.4 (2)
C19A—C17A—C18A—C13A	−179.9 (2)	C16B—C17B—C18B—C13B	−0.5 (3)
N14A—C13A—C18A—C17A	0.1 (4)	C19B—C17B—C18B—C13B	179.9 (2)
N12A—C13A—C18A—C17A	178.7 (2)	N14B—C13B—C18B—C17B	−1.2 (4)
C18A—C17A—C19A—C20A	33.6 (4)	N12B—C13B—C18B—C17B	179.6 (2)
C16A—C17A—C19A—C20A	−147.5 (3)	C18B—C17B—C19B—C20B	−32.0 (4)
C18A—C17A—C19A—N23A	−146.5 (2)	C16B—C17B—C19B—C20B	148.5 (3)
C16A—C17A—C19A—N23A	32.4 (3)	C18B—C17B—C19B—N23B	148.8 (2)
N23A—C19A—C20A—N21A	0.7 (3)	C16B—C17B—C19B—N23B	−30.8 (3)
C17A—C19A—C20A—N21A	−179.4 (3)	N23B—C19B—C20B—N21B	−1.4 (3)
N23A—C19A—C20A—C24A	−175.8 (3)	C17B—C19B—C20B—N21B	179.3 (3)
C17A—C19A—C20A—C24A	4.1 (5)	N23B—C19B—C20B—C24B	175.7 (3)
C19A—C20A—N21A—C22A	−0.2 (3)	C17B—C19B—C20B—C24B	−3.6 (5)
C24A—C20A—N21A—C22A	176.9 (2)	C19B—C20B—N21B—C22B	0.9 (3)
C20A—N21A—C22A—N23A	−0.4 (3)	C24B—C20B—N21B—C22B	−176.7 (2)
C20A—N21A—C22A—S31A	−179.91 (19)	C20B—N21B—C22B—N23B	−0.1 (3)
N21A—C22A—N23A—C19A	0.8 (3)	C20B—N21B—C22B—S31B	179.12 (19)
S31A—C22A—N23A—C19A	−179.7 (2)	N21B—C22B—N23B—C19B	−0.8 (3)
C20A—C19A—N23A—C22A	−0.9 (3)	S31B—C22B—N23B—C19B	−179.9 (2)
C17A—C19A—N23A—C22A	179.1 (2)	C20B—C19B—N23B—C22B	1.3 (3)
C19A—C20A—C24A—C25A	36.6 (4)	C17B—C19B—N23B—C22B	−179.3 (2)
N21A—C20A—C24A—C25A	−139.5 (3)	C19B—C20B—C24B—C25B	−37.8 (4)
C19A—C20A—C24A—C29A	−145.1 (3)	N21B—C20B—C24B—C25B	138.9 (3)
N21A—C20A—C24A—C29A	38.9 (4)	C19B—C20B—C24B—C29B	143.4 (3)
C29A—C24A—C25A—C26A	0.5 (4)	N21B—C20B—C24B—C29B	−39.9 (4)
C20A—C24A—C25A—C26A	178.9 (2)	C29B—C24B—C25B—C26B	−0.1 (4)
C24A—C25A—C26A—C27A	0.5 (4)	C20B—C24B—C25B—C26B	−179.0 (2)
C25A—C26A—C27A—F30A	−179.6 (2)	C24B—C25B—C26B—C27B	−0.3 (4)
C25A—C26A—C27A—C28A	−0.5 (4)	C25B—C26B—C27B—C28B	0.1 (5)
F30A—C27A—C28A—C29A	178.5 (2)	C25B—C26B—C27B—F30B	179.1 (3)
C26A—C27A—C28A—C29A	−0.5 (4)	F30B—C27B—C28B—C29B	−178.6 (3)
C27A—C28A—C29A—C24A	1.6 (4)	C26B—C27B—C28B—C29B	0.4 (5)
C25A—C24A—C29A—C28A	−1.6 (4)	C27B—C28B—C29B—C24B	−0.9 (4)
C20A—C24A—C29A—C28A	180.0 (2)	C25B—C24B—C29B—C28B	0.7 (4)
N23A—C22A—S31A—C32	−10.0 (6)	C20B—C24B—C29B—C28B	179.6 (3)
N21A—C22A—S31A—C32	169.4 (6)	N23B—C22B—S31B—C32B	−16.4 (3)
N23A—C22A—S31A—C32A	11.0 (5)	N21B—C22B—S31B—C32B	164.5 (2)

### Hydrogen-bond geometry (Å, °)

**Table:**

*D*—H···*A*	*D*—H	H···*A*	*D*···*A*	*D*—H···*A*
N12A—H12A···F9A	0.88	2.15	2.605 (2)	112
N21A—H21A···O11A^i^	1.06	1.89	2.813 (2)	143
N12B—H12B···F9B	0.88	2.15	2.608 (2)	112
N21B—H21B···O11B^ii^	0.96	1.86	2.796 (2)	165

Symmetry codes: (i) −*x*+1, *y*+1/2, −*z*+1/2; (ii) −*x*, *y*+1/2, −*z*+1/2.
